# The Delta and Omicron Variants of SARS-CoV-2: What We Know So Far

**DOI:** 10.3390/vaccines10111926

**Published:** 2022-11-14

**Authors:** Vivek P. Chavda, Rajashri Bezbaruah, Kangkan Deka, Lawandashisha Nongrang, Tutumoni Kalita

**Affiliations:** 1 Department of Pharmaceutics and Pharmaceutical Technology, L M College of Pharmacy, Ahmedabad 380008, Gujarat, India; 2 Department of Pharmaceutical Sciences, Faculty of Science and Engineering, Dibrugarh University, Dibrugarh 786004, Assam, India; 3 NETES Institute of Pharmaceutical Science, Mirza, Guwahati 781125, Assam, India; 4 Girijananda Chowdhury Institute of Pharmaceutical Science, Azara, Guwahati 781017, Assam, India

**Keywords:** SARS-COV-2, Delta variant, Delta plus variant, vaccination, vaccine efficacy, variant of concern, Omicron variant

## Abstract

The world has not yet completely overcome the fear of the havoc brought by SARS-CoV-2. The virus has undergone several mutations since its initial appearance in China in December 2019. Several variations (i.e., B.1.616.1 (Kappa variant), B.1.617.2 (Delta variant), B.1.617.3, and BA.2.75 (Omicron variant)) have emerged throughout the pandemic, altering the virus’s capacity to spread, risk profile, and even symptoms. Humanity faces a serious threat as long as the virus keeps adapting and changing its fundamental function to evade the immune system. The Delta variant has two escape alterations, E484Q and L452R, as well as other mutations; the most notable of these is P681R, which is expected to boost infectivity, whereas the Omicron has about 60 mutations with certain deletions and insertions. The Delta variant is 40–60% more contagious in comparison to the Alpha variant. Additionally, the AY.1 lineage, also known as the “Delta plus” variant, surfaced as a result of a mutation in the Delta variant, which was one of the causes of the life-threatening second wave of coronavirus disease 2019 (COVID-19). Nevertheless, the recent Omicron variants represent a reminder that the COVID-19 epidemic is far from ending. The wave has sparked a fervor of investigation on why the variant initially appeared to propagate so much more rapidly than the other three variants of concerns (VOCs), whether it is more threatening in those other ways, and how its type of mutations, which induce minor changes in its proteins, can wreck trouble. This review sheds light on the pathogenicity, mutations, treatments, and impact on the vaccine efficacy of the Delta and Omicron variants of SARS-CoV-2.

## 1. Introduction

**Figure 1:**
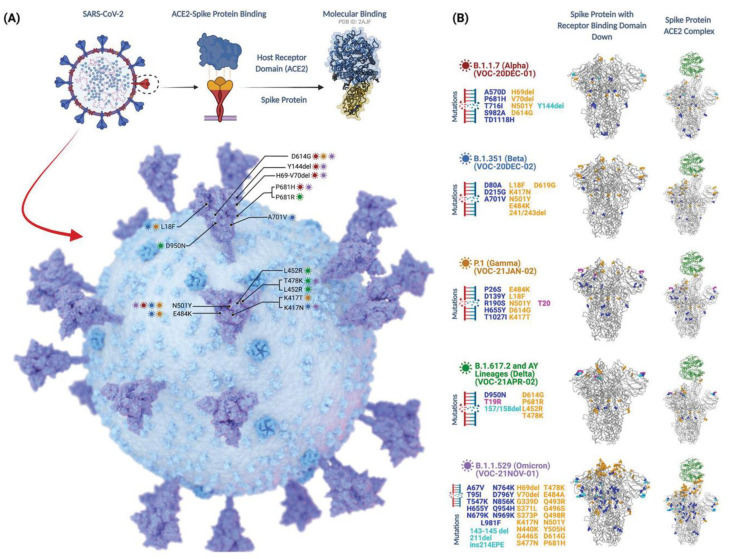
Summary of SARS-CoV-2 mutants/variants: (**A**) illustration of S-protein–ACE2 binding, location of the frequently detected mutations within the spike region of SARS-CoV-2; (**B**) detailed visualization of mutations that are found within the genomic structure of current VoC lineages (adopted under CC BY 4.0 License from [50]).

**Figure 2:**
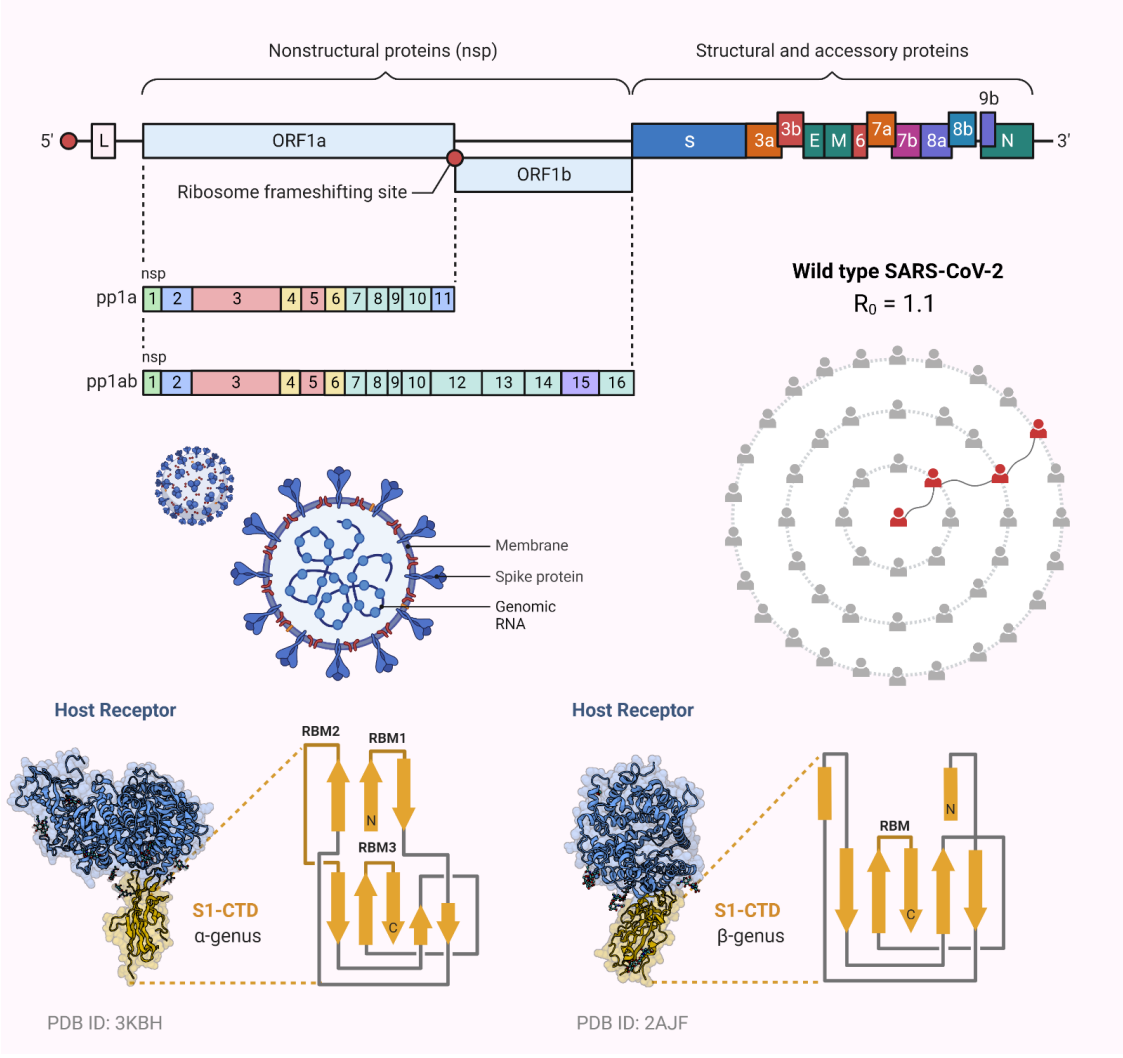
Schematic diagrams of the SARS-CoV-2 genome with structure. Reproduction number of wild-type SARS-CoV-2 is 1.1 which is still higher in the emerging variants of SARS-CoV-2. The receptor-binding motif mainly interacts with host receptor for viral host entry (created with Biorender.Com).

In late December 2019, Wuhan, a small town in China, was affected by several pneumonia cases, which were indicated by dry cough, fever, fatigue, and sometimes intermittent gastrointestinal symptoms. The disease rapidly spread from one person to another, from town to town, and later throughout the whole Chinese nation and, ultimately, to the whole world. On 11 February 2019, the WHO named this disease COVID-19, which is from the family of coronaviruses. Structurally, the coronavirus family (CoV) consists of a single-stranded RNA virus, which is responsible for the transmission of infections from one person to another (human coronavirus, hCoV). The virus is commonly recognized as severe acute respiratory syndrome-CoV-2 (SARS-CoV-2), which is a member of the betacoronavirus genus [1,2,3,4,5]. Due to the fast spread of this dreadful disease throughout the whole world, the WHO announced a global pandemic on 11 March 2019 [6]. This novel coronavirus has spread to all corners of the earth and has led to the deaths of nearly one million people around the globe [7]. Moreover, the most vulnerable ones identified are the immunocompromised patients, and it is more likely for viral infections to progress to severe disease in these patients [8]. Researchers have found that the main receptor through which the SARS-CoV-2 binds to form a complex is the angiotensin-converting enzyme-2 (ACE2), a homolog of ACE, which allows the virus to enter the host cell [9]. Due to viral mutations in the disease, ample research efforts toward a vaccine and therapeutic development against SARS-CoV-2 are being made, not only for humans but also for animals, to halt this unwanted phase [10,11,12,13,14,15]. Much like any other RNA virus, SARS-CoV-2 is rapidly mutating, with new variations arising as long as transmission continues (Figure 1 and Figure 2). Figure 1A demonstrates the key mutations of the viral spike proteins of different variants of concern (VOCs) that majorly interact with ACE2, while Figure 1B gives detailed visualization of those mutations. Some alterations in the Omicron genome are the same as those found in other VOCs, and they are responsible for enhanced transmission rates, immunoescape qualities, and a greater likelihood of contracting the illness due to a stronger affinity for the ACE2 receptors [16]. At the 5′ end are two large ORFs, ORF1a and ORF1b, covering more than two-thirds of the genome (Figure 2) where mutations are observed in different VOCs [17,18]. If a variant provides a selective advantage to the virus, it may become more prevalent. SARS-CoV-2 variants can be grouped into three different categories, i.e., the variants of interest (VOI), the variants of high consequence, and VOC. Since the start of the pandemic, the WHO has announced several variants as VOCs; they include strains B.1.1.7 (Alpha), B.1.351 (Beta), P.1 (Gamma), B.1.617.2 (Delta), and most recently, the B.1.1.529 (Omicron) variant, each of which has different transmissibility, evasive nature, and neutralization capabilities [19,20]. Recently, the Indian Ministry of Health officially provided information on the genome sequencing of 10,787 specimens that were collected from the 18 states of India. Almost 90% of the samples are positive with the Alpha variant, 4% with the Beta variant, 1% with the Gamma variant, and the remaining 5% of the samples were positive with variants such as B.1.617, B.1.616.1 (Kappa), and B.1.617.3 [21]. The Alpha variant B.1.1.7, which is originally a UK strain, is prevalent in Punjab, as all the 336 samples tested were positive for the Alpha variant. In 206 Maharashtra samples, a novel VOC contained two alterations, E484Q and L452R, which were evident [22,23]. B.1.617, informally known as the Indian variant, was initially detected on 1 December 2020; however, it did not become prevalent until February 2021. This mutant SARS-CoV-2 strain that was formerly detected in India (Figure 1) comprises three main varieties, B.1.616.1 (Kappa-VOI), B.1.617.2 (Delta-VOC), and B.1.617.3 [24]. All the identified Indian strains of SARS-CoV-2 have a unique genetic make-up. The WHO declared the Delta variant a VOC on 15 June 2021. It first appeared in Maharashtra (India) in December 2020. However, its devastating impact was observed in late April 2021, when more than 30,000 cases were reported daily in New Delhi (India) [25,26,27]. 

The variant spread like wildfire from India, and in current circumstances, it has caused a devastating new wave throughout the globe. Globally, there are more than 11 million incidences of the Delta variant, and the UK has reported the largest number of COVID-19 Delta variant cases, i.e., around one million, as of 19 December 2021 [28]. In 2021, the WHO made a statement that the Delta strain was sweeping the globe at breakneck speed, which led to a new surge in cases and deaths [29]. More than 0.1 million sequences of the Delta variant samples have been detected since the lineage’s identification, and the strain has been reported in at least 148 nations [30]. The Delta variant gained much attention in England, but the good news is that it is believed to lack the 484K/Q mutation, one that has recently been linked to resistance to vaccination [31,32]. It has two escape alterations, E484Q and L452R, as well as other mutations; the most notable of these is P681R, which is expected to boost infectivity [33]. As per studies conducted in the UK, the Delta symptoms are slightly different from those of other strains, although this does not always indicate serious symptoms. Symptoms such as a sore throat, headache, runny nose, and fever are common, but olfactory dysfunction and cough are not as common. Some severe symptoms associated with Delta include serious gastrointestinal issues, hearing impairment, and tissue death owing to blood clots [34]. Some study reports from Canada as well as Scotland concluded that the rate of hospitalization is higher in the case of Delta-infected patients compared with patients infected with other variants [35]. Moreover, B.1.617 has improved resistance to neutralizing antibodies by both immunization and native COVID-19 but not to the point where the vaccine is rendered useless [24]. To mitigate the current pandemic situation, vaccines are playing vital roles by showing remarkable safety and efficacy. Nevertheless, they are not without flaws, as breakthrough infections have been reported owing to the improved resistance of the different variants over neutralizing antibodies. Breakthrough infections are often less common in fully vaccinated people. The same applies in the case of Delta variant infections. Unvaccinated individuals tend to be the major source of concern. People who have been fully vaccinated but have developed Delta breakthrough infections can also transfer the virus to others, although they appear to disseminate the virus for a short period. The current rapid increase in Delta variant instances has been attributed to low routine immunization in several areas. In such cases, Delta posed a significant concern to the world’s poorest countries, which have limited or no access to vaccinations [36].

Delta variants appeared to transmit at a higher rate than other VOCs [25]. However, in November 2021, the WHO declared another variant as a VOC, and this variant is known as the “Omicron” [37]. It was initially discovered in South Africa in mid-November 2021 [38]. On 31 August 2022, the Director-General of the WHO stated at a media briefing that, in addition to their predecessors, Omicron subvariants are more transmissible, and further variants will likely be even more dangerous and transmissible and had advised people to get vaccinated and also receive a booster if needed [39]. The Omicron variant had the highest number of mutations (50) throughout its genome out of the five VOCs investigated. Researchers have found that the Omicron variant’s infectivity and immune escape are relatively greater than those of the other four VOCs because of the high mutation load it carries [40]. The Omicron variant has 32 spike protein mutations, double the Delta variant [41]. Notably, more than 15 of these alterations seem to be present in the receptor-binding domain (RBD), with the main goal of neutralizing antibodies (NAbs) [42], which are significantly more frequent than those found in other VOCs [43,44]. In addition to the critical genetic alterations that have been reported in the spike protein of the Omicron variant, similar mutations have also been observed in the other VOCs and VOIs. These mutations include H655Y, K417N in Gamma; D614G, Δ69–70, P681H, and N501Y in Alpha; T478K mutation in Delta; and K417N in Beta [45]. Omicron’s BA.5 strain is currently the most contagious and dominant subvariant globally. Nevertheless, this subvariant is not considered more severe than the other subvariants, and the COVID-19 vaccine is considered safe and effective against serious infections. However, global spikes in cases can strain health services to the breaking point [46,47]. A recent report indicated that an emerging sublineage, known as BA.2.75, is growing in prevalence in India. Nevertheless, as of 19 July 2022, BA.2.75 had been reported in at least fifteen nations [48]. At present, public health restrictions have been loosened in many countries following the third consecutive year of the global pandemic. Vaccination or natural infection has now made most of the population globally immune to the deadly SARS-CoV-2, but new variants will likely emerge, triggering local outbreaks and affecting prevention and treatment in unpredictable ways. This review interprets the global impact of the Delta and Omicron variants outbreaks, including their pathogenicity, mutation, therapeutics, and influence on vaccine efficacy [49].

## 2. Mutation and Pathogenesis of Delta and Omicron Variants

SARS-CoV-2 is a disease that is constantly mutating, leading to further new variants. One such variant is the Delta variant, which was mentioned earlier to be among the most prevalent strains of SARS-CoV-2 to exist to date. The variant is known to have several mutations at 22 different amino acids of different genes, including the ORF, S, M, and N (Table 1) [51]. From these genes, the S protein of the virus plays an important role by offering adhesion to the host cell, which thereby allows access to the cells [52]. The S protein is the principal target of the COVID-19 vaccine; likewise, the majority of the serum-neutralizing antibody responses induced through spontaneous SARS-CoV-2 infections are concentrated on the RBD of the S protein [12,53,54]. The spike protein is composed of S1 and S2 subunits; S1 interacts with the ACE2 receptor, and S2 assists in cellular integration and viral fusion [11,55]. After entering the cells, the immune cells of the body flag the S protein as foreign, which will further lead to the generation of antibodies by the B cells, and these antibodies adhere to the virus and eradicate it. If mutations occur in the S protein, which is not recognized by first-wave antibodies, the developed immunity against the original strain (followed by exposure or vaccination) may be found to be ineffective [41]. Due to this, it becomes increasingly difficult for the immune system to recognize and bind to S proteins as they mutate [34,56].

**Figure 3:**
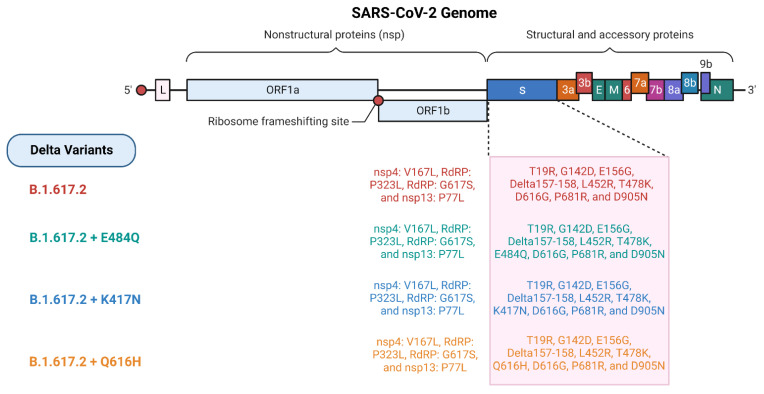
Different lineages of SARS-CoV-2 Delta variants (created with Biorender.com).

There are 4 different lineages of Delta variants (Figure 3), while 12 different lineages of Delta plus variants have been recorded. The Delta variant of SARS-CoV-2 comprises nine S-protein mutations, namely T19R, G142D, L452R, D614G, T478K, D960N, P681R, and E156G, with deletions at 157 and 158 positions. Nevertheless, the RBD also includes multiple mutations such as mutations in the “antigenic supersite” region of the N-terminal domain and also at the furin cleavage site [34]. The S-protein mutations at positions L452R and P681 are most notable, affecting the antibody binding. Among these, the mutation of L452 replaces an amino acid, “arginine”, at 452 positions for another amino acid called “leucine”, whereby the interaction between the S protein and ACE2 receptor is enhanced. However, as residue L452 is found in the RBD’s hydrophobic plaques in the S protein, it does not make any direct contact with the ACE2 receptor [57]. The ability of this novel S protein to evade the immune system allows better adhesion to human cells, leading to more aggressive infection [58]. Moreover, the P681R mutation is near the S protein’s furin cleavage site and enhances the number of basic residues in the substandard S protein’s furin cleavage site [59]. A study on hamsters concluded that the mutation of P681R does not increase infectivity but shows more pathogenicity compared with the parent SARS-CoV-2 virus [60,61,62]. Furthermore, it can reduce neutralizing antibodies partially. According to a “pseudovirus neutralization test”, the D614G/P681R mutation was shown to target the RBD of the S protein by exhibiting moderate resistance (1.2–1.5 times) to three monoclonal antibodies (mAbs) [63]. In addition to enhancing intercellular fusion, P681R also promotes furin-mediated S-protein cleavage [59,64].

**Table 1:** Characteristic mutations of Delta variants [65].

Protein	Amino Acid Mutation	Delta Variant Lineages	Mutation Impact
ORF1a	T3255I	B.1.617.2; B.1.617.2 plus Q613H; B.1.617.2 plus E484Q; B.1.617.2 plus K417N	The ability of viruses to adapt to internal interactions in host cells, such as replicating and transcribing viral genomes and budding by cellular exocytosis, as well as external interactions with host cells, such as recognizing a cell surface receptor, attaching to the host receptor, and fusing with cellular membranes [66].
ORF1b	P314L	B.1.617.2; B.1.617.2 plus E484Q; B.1.617.2 plus K417N; B.1.617.2 plus Q613H	
	G662S	B.1.617.2; B.1.617.2 plus E484Q; B.1.617.2 plus K417N; B.1.617.2 plus Q613H	
	P1000L	B.1.617.2; B.1.617.2 plus E484Q; B.1.617.2 plus K417N; B.1.617.2 plus Q613H	
S	T19R (NTD)	B.1.617.2; B.1.617.2 plus Q613H; B.1.617.2 plus E484Q; B.1.617.2 plus K417N	Increase the immune evasion [67].
	G142D (NTD)	B.1.617.2; B.1.617.2 plus Q613H; B.1.617.2 plus E484Q; B.1.617.2 plus K417N	
	E156G (NTD)	B.1.617.2; B.1.617.2 plus Q613H; B.1.617.2 plus E484Q; B.1.617.2 plus K417N	
	del157/158 (NTD)	B.1.617.2; B.1.617.2 plus Q613H; B.1.617.2 plus E484Q; B.1.617.2 plus K417N	
	L452R (RBD)	B.1.617.2; B.1.617.2 plus E484Q; B.1.617.2 plus K417N; B.1.617.2 plus Q613H	Impacts neutralization by monoclonal antibodies [68].
	T478K (RBD)	B.1.617.2; B.1.617.2 plus Q613H; B.1.617.2 plus E484Q; B.1.617.2 plus K417N	Aids in antibody emigration [69].
	D614G (SD2)	B.1.617.2; B.1.617.2 plus Q613H; B.1.617.2 plus E484Q; B.1.617.2 plus K417N	Enhances the infectivity and density of virion spikes [70].
	P681R (furin-cleavage site)	B.1.617.2; B.1.617.2 plus Q613H; B.1.617.2 plus E484Q; B.1.617.2 plus K417N	It improved the full-length spike’s cleavage to S1 and S2, which increased infection through cell surface penetration [64,71].
	D950N (S2 region)	B.1.617.2; B.1.617.2 plus Q613H; B.1.617.2 plus E484Q; B.1.617.2 plus K417N	It might alter the spike protein’s structure to make it better able to shift its form and unite with human cells [72].
	Q613H	B.1.617.2 plus Q613H	Upsurges transmissibility andPathogenicity [73,74].
	K417N	B.1.617.2 plus K417N	Combining the K417N mutation with N501Y eliminated the antibody effect [75].
	E484Q	B.1.617.2 plus E484Q	Exceedingly resistant to neutralization by mAb [76].
ORF3a	S26L	B.1.617.2; B.1.617.2 plus Q613H; B.1.617.2 plus E484Q; B.1.617.2 plus K417N	This mutation modifies Orf3a protein dynamics, protein disorder parameters, and secondary structure [77].
M	I82T	B.1.617.2; B.1.617.2 plus Q613H; B.1.617.2 plus E484Q; B.1.617.2 plus K417N	Crucial for viral assembly, it may also affect glucose transport and decrease type I and type III interferon production, which severely reduces the innate immune response [78].
ORF7a	V82A	B.1.617.2; B.1.617.2 plus Q613H; B.1.617.2 plus E484Q; B.1.617.2 plus K417N	It restricts host immune suppression with interferon antagonism and might be responsible for causing the expression of pro-inflammatory cytokines [79,80].
	T120I	B.1.617.2; B.1.617.2 plus Q613H; B.1.617.2 plus E484Q; B.1.617.2 plus K417N	
ORF8	S84L	B.1.617.2; B.1.617.2 plus Q613H; B.1.617.2 plus E484Q; B.1.617.2 plus K417N	The virus evades the immune system by altering its binding affinity with IRF3 and disrupting chromatin regulation, which speeds up reproduction [81,82].
	del119/120	B.1.617.2; B.1.617.2 plus Q613H; B.1.617.2 plus E484Q; B.1.617.2 plus K417N	
N	D63G	B.1.617.2; B.1.617.2 plus Q613H; B.1.617.2 plus E484Q; B.1.617.2 plus K417N	Alteration in secondary structure [83].
	R203M	B.1.617.2; B.1.617.2 plus Q613H; B.1.617.2 plus E484Q; B.1.617.2 plus K417N	
	D377Y	B.1.617.2; B.1.617.2 plus Q613H; B.1.617.2 plus E484Q; B.1.617.2 plus K417N	

S = spike protein, ORF= open reading frames, N= nucleocapsid, M= membrane protein.

Omicron, the most recent SARS-CoV-2 variant, was identified in South Africa, which has also had a huge impact around the world and is known to be a more dominant variant than any other variant due to its high infectivity and antibody evasion. The Omicron variant became known as the fifth VOC by the WHO on 26 November 2021. B1.1.529, the first Omicron variant, was originally discovered in Botswana, and from there, the variant spread across the entire South African province and beyond [37]. Unlike other SARS-CoV-2 variants, Omicron largely spares the olfactory function, which may be due to its hydrophobicity and also its alkalinity, which is even more than the Wuhan strain. In this way, Omicron may exhibit a decreased solubility in mucus, thereby reducing olfactory epithelial infections [84]. The Omicron variant is known to have around 60 mutations, with 15 alterations in the RBD and 36 alterations in the S protein; therefore, it has the most mutation sites of all known coronavirus variants [85,86,87]. In addition, there are five lineages of the Omicron variant, i.e., BA.1 to BA.5. Although BA.1 was the original predominant strain, BA.2 is rapidly overtaking it in many countries. At most, there are a few hundred cases of BA.3, which has very limited transmissibility. Pango lineages BA.4 (22A) and BA.5 (22B) were recently discovered and are on the rise, which is different from Pango lineages BA.2.12.1. There are 57 mutations in the BA.2 lineage, 31 of them in the S protein, with a significant difference in the N-terminus between BA.1 and BA.2. ACE2, which is the host receptor of the S protein, increases infectivity and escapes the neutralizing antibodies induced by vaccines when bound. As a result, considerable research has been carried out on those mutations that affect the RBD of the S protein. Twelve mutations, namely G339D, S477N, N501Y, S373P, T478K, S375F, K417N, N440K, Y505H, E484A, Q493R, Q498R, and R346K, were discovered in one of these members of the group, namely BA.1.1, while S371L, G446S, and G496S were associated with BA.1 only. Besides S371F and R408S, BA.2 also shares common mutations T376A and D405N with BA.3. Similarly, previous variants have also displayed such kinds of mutations that are known to increase antibody sensitivity and resistance. In Beta (B.1.351) and Gamma (P.1) variants, alterations in N501, K417, and E484 residues may play a role in the vaccine-induced neutralization. The S protein (subunits: S1-S2) promotes viral entrance into the host cell on interaction with the host receptors. Viral entry into host cells requires cathepsin L or type II transmembrane serine protease (TMPRSS2) and furin, which breaks down the two subunits of the S protein with three alterations in the furin cleavage site; the mutations are namely N679K, P681H, and H655Y. Compared with the second wave, the third wave shows significantly greater levels of infection and transmission. According to the early trend data in India, compared with other Omicron-affected nations (SA, France, US, UK, and Italy), the third wave looks to be mostly driven by Omicron, although hospitalizations and infection-related casualties are estimated to be fewer than in other Omicron-affected nations based on evidence from other nations [45]. 

**Figure 4:**
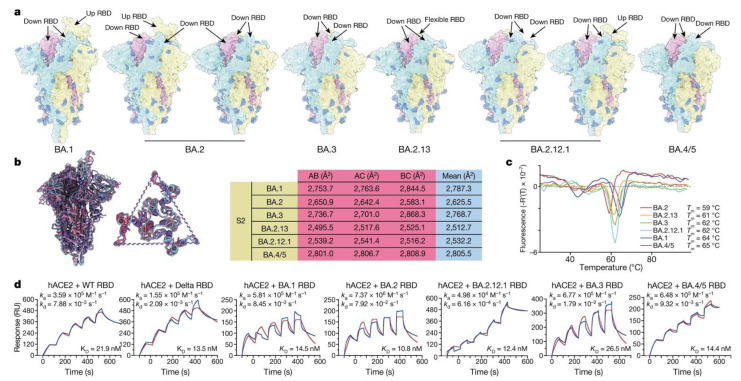
Structural and receptor-binding characteristics of Omicron subvariants: (**a**) surface representation of S-trimers of BA.1, BA.2, BA.3, BA.2.13, BA.2.12.1, and BA.4/BA.5 (BA.4/5) variants; (**b**) structural interpretation and functional verification of the stability of the spike protein of BA.1, BA.2, BA.3, BA.2.13, BA.2.12.1, and BA.4/BA.5 variants. Left, superimposed structures of spike protein and the S2 domains of BA.1 (purple), BA.2 (red), and BA.4/BA.5 (blue). The binding surface areas between S2 subunits of the variants are calculated in the table on the right; (**c**) thermofluor analysis for these Omicron variants. Analyses were performed as biological duplicates; (**d**) binding affinities of RBDs of Omicron variants for hACE2 measured by SPR. Analyses were performed as biological duplicates. (Figure 4 is adapted from Yunlong Cao et al. (2022) [89] via CC by 4.0 license.).

The T4 fibritin trimerization domain, Gly-Ser-Ala-Ser (GSAS), and 6P mutations are present in all the spike proteins (S-trimers) of the Omicron lineage (BA.1, BA.2, BA.3, BA.2.12.1, BA.2.13, and BA.4/BA.5) for enhanced stability [88]. The differences between the Omicron sublineages were revealed by a study using cryo-EM conducted by Yunlong Cao et al. [89]. According to that study, in contrast to the BA.1 S-trimer, which is stable in an open conformation with one “up” RBD and two “down” RBDs, the BA.2 and BA.2.12.1 spikes exhibit two conformational states that correspond to a closed form with all three RBDs in the down configuration and an open form with one RBD in the up position. In addition to BA.2 and BA.2.12.1, BA.2.13 notably contained one RBD that was visibly disrupted, showing a stochastic shift that supports structural variability in the S-trimers of BA.2 sublineages (Figure 4a). The study also showed that the N658S mutation, which may be correlated with the more closed RBD configurations of the BA.4/BA.5 S-trimer, was initially present in early BA.4/BA.5 lineages but later vanished due to the poor exposure of this variant. Notably, in the region created by the three copies of S2, S-trimers from the BA.2 sublineage had considerably fewer compact topologies (Figure 4b). Contrarily, BA.1, BA.3, and BA.4/BA.5 spikes have more buried areas between the S2 subunits and a rather compact inter-subunit organization (Figure 4b). Thermal stability assays by the researchers confirmed that S-trimers from BA.2 sublineages were the least stable of these variations, in agreement with structural data, which may lead to an improved fusion efficiency (Figure 4c). Compared with the other Omicron subvariants, the BA.4/BA.5 S-trimer displayed a lower binding affinity for hACE2; nevertheless, this assessment may not be accurate due to the extra N658S mutation [89]. Except for the BA.3 RBD, which displayed a reduced affinity comparable to that of the original WT strain, the binding affinities of the RBDs of the Delta (B.1.617.2) and the circulating Omicron subvariants for ACE2 were comparable. Additionally, compared with the other Omicron subvariants, the BA.2 subvariants showed somewhat higher binding affinities for hACE2 (Figure 4d). Additionally, research using molecular dynamics simulations shows that the absence of G496S in BA.2 sublineages resulted in the restoration of the hydrogen bond with hACE2 K353, enhancing their ability to bind. The hydrophilic interaction between BA.3 spike (S446) and hACE2 Q42 was nonetheless disrupted by local conformational disturbance at the spike residues 444–448; this is likely due to the single mutation G446S rather than the double mutations G446S and G496S. Interestingly, the hACE2 binding activity is decreased by the F486V mutation in the BA.4/BA.5 spike due to the decreased hydrophobic interaction [89]. Table 2 shows the characteristic mutations of the omicron variant.

## 3. Omicron Variant vs. Delta Variant

Of all the variants examined so far, Omicron has been detected as the variant with the highest number of alteration sites. Approximately 60 substitutions, insertions, and deletions have been studied in this variant, whereas the Delta variant has only around 22 mutations [86]. These mutations may be associated with an increased risk of transmission and reinfection, as well as reduced vaccination effectiveness [26]. The researchers from the “LKS Faculty of Medicine at the University of Hong Kong” discovered that Omicron multiplies 70-fold faster in the human bronchus when compared to the Delta variant and the classic SARS-CoV-2 variant, which may help to explain why it spreads more rapidly than earlier variants [16,26,99,100,101]. The spike RBD is the legitimate viral entity that identifies the ACE2 receptor and promotes viral entry. The RBD of Omicron has 15 mutations, whereas only L452R and T478K variations are prevalent in the RBD of the Delta variant. Of these changes, a group of residues appears to be located near the bound ACE2 receptor [86]. A computational study was conducted in a study by Kumar et al. to analyze the Delta and Omicron variants. It was discovered that the Delta variant’s RBD site underwent significant modifications, which increased the association of Omicron and ACE2, possibly leading to a faster transmission rate [26]. Several mutations led to a higher binding affinity for human ACE2 based on in silico studies: Q493R, N501Y, S375F, S371L, S373P, T478K, and Q498R. Furthermore, a wide variety of lipophilic amino acids, namely phenylalanine and leucine, are found in the RBD as well as in the entire S protein of Omicron when compared to Delta [26]. Moreover, it is possible that the TMPRSS2 route of cell entry, along with the high membrane fusion capacity, is required for the rapid induction of anosmia in SARS-CoV-2 variant Delta, and this route may be the only one able to cause rapid anosmia in COVID-19 due to its enhanced infectivity, while the Omicron variant uses the less efficient endosomal route, thus appearing in the failure to induce frequent anosmia [84]. Uncertainty surrounds the question of whether Omicron infection results in more severe complications than infections brought on by other variants, such as the Delta variant. All COVID-19 variants, including the globally widespread Delta variants, can cause serious illness or death [102]. Omicron variant infections continue to climb globally, with a total of 2780 confirmed cases as of 19 December 2021 [103]. However, researchers are investigating to find out if the Omicron variant affects COVID-19 vaccination efficiency. Despite inadequate data, the WHO feels it is acceptable to assume that the currently available vaccinations provide some defense against severe complications and morbidity. The currently available vaccines produce a reduced level of neutralizing antibodies against the Omicron variant of SARS-CoV-2, suggesting that the geriatric population is at a greater infection risk against this variant, and it is advised to get fully vaccinated and can even receive a booster dose when recommended [104].

Quick vaccine development has been critical in combating the continuing COVID-19 epidemic. Nevertheless, access issues persist, new infections emerge, and evolving variations provide a higher danger [105]. Generating antiviral medicines is thus a top goal for COVID-19 therapy. Evaluating viral transmission, variant emergence, and mutation rates are crucial for creating successful treatments and vaccines [106,107]. As health officials throughout the world strive to vaccinate their populations in order to achieve herd immunity, the problems identified suggest that COVID-19 treatments are still required to act alongside vaccinations [108]. Outpatients with mild to moderate COVID-19 who are at risk of hospitalization owing to comorbidities or other circumstances are frequently treated with neutralizing anti-SARS-CoV-2 monoclonal antibodies [109]. The introduction of novel variants with lower susceptibility to neutralization by vaccine-induced antibodies is perhaps most concerning, as these variants pose the greatest danger to the protective effect of existing vaccinations [110]. Since viral mutations generally demonstrate mutations in the viral spike protein, which is the target for most of the early diagnostic tests as well as neutralizing antibodies, understanding viral mutation is very important for the emerging viral variants. The consequences of novel variations on viral transmissibility, illness severity, reinfection rates (i.e., escape from natural immunity), and vaccine efficacy are four major issues raised by their development (i.e., escape from vaccine-induced immunity) [51].

## 4. Vaccine Efficacy

### 4.1. Delta Variants Influence Vaccine Efficacy

The Delta variant is known for its high infectivity. According to research, this strain’s infectivity is 97% to 100% higher than the original epidemic strain, i.e., the Wuhan strain [111]. In addition, the mutation of the RBD variants reduces the immune response of the host’s cells, which has led to numerous reports of breakthrough infections occurring after complete immunization [112,113,114]. A large number of vaccines authorized and delivered across the world had completed phase II/III clinical research before the Delta variant outbreak, and their effectiveness was mostly confined to individuals exposed to other VOCs of novel coronavirus. Thus, the outbreak of the Delta variant called into question their ability to provide adequate defense. Nevertheless, studies related to the evaluation of vaccine efficacy for the Delta variant have been widely reported. Studies have also supported the concept of a “heterologous prime–boost COVID-19 vaccine strategy”, in which vaccines from different platforms can be used as an effective immunization strategy against COVID [115].

ChAdOx1 or AZD1222, an adenovirus vectored vaccine designed by Oxford-AstraZeneca, was one of the most commonly used preventive measures during the COVID pandemic, and a two-dose regimen of the vaccine yielded approximately 70% efficacy in the clinical trials. The neutralizing titer of this vaccine fell by 2.5–9.0 times more against the Delta virus variant than against the Alpha variant [67]. However, Covishield, the Indian version of ChAdOx1 (developed by the Serum Institute of India), displayed a 3.28-fold drop in neutralizing titer against the Delta variant followed by a second dose when compared with D614G (a spike protein mutation) [116]. The immune serum geometric mean titer, following the first dose, was less than that after the double dose, and the degree of decline was greater, which suggests the significance of the second dosage [117,118]. Additionally, Ad26.CoV2-S (the Johnson and Johnson adenovirus vector vaccine) reported cross-neutralization data. The neutralizing titer against the Delta variant was reduced by 1.72–3.40-fold after one dose of Ad26.CoV2-S when compared with WT/ Alpha [119,120]. Similarly, the mRNA vaccine developed by Pfizer, BNT162b2, showed a 1.41–8.40 time decrease in the neutralizing titer against the Delta variant in comparison with WT/Alpha [121]. The immune serum following two doses of ChAdOx1 and BNT162b2 was evaluated in a series of trials, and the neutralizing potential of ChAdOx1 against the Delta virus was found to be slightly lower than that of BNT162b2 [24,67]. In another study, an evaluation of the immune serum using the pseudovirus following two injections of BNT162b2 showed that the neutralizing antibody titer was reduced by 2.83–11.30-fold against the Delta variant relative to WT/D614G, which is identical to the results attained by the live virus [24,122]. Another widely used mRNA vaccine, mRNA-1273, was also reported with cross-neutralization data. The Delta variant had a greater decrease in the neutralizing titer (2.10–3.80 fold), compared with D614G than either the Alpha or Beta variants (1.20 fold) but still less than the decline seen with D614G. (2.20–8.40 times) [123]. Additionally, BBV152 (an Indian inactivated vaccine) had a 2.7-fold lower immunological serum-neutralizing titer against Delta live virus than D614G [124]. Furthermore, CoronaVac, the inactivated vaccine created by Sinovac in China, reported a reduction in the neutralizing titer against the Delta virus by 31.64-fold when compared with the WT, which was more than the reduction in that against Alpha and Beta variants utilized in the same study (17.35 and 22.11 times, respectively) [125]. One dose of BNT162b2 or ChAdOx1 may provide equivalent immunity against the Delta variant in those who had priorly contracted COVID-19 to those who had been vaccinated with three doses of CoronaVac [126].

In another trial, one dose of the FINLAY-FR-1A vaccine generated long-term memory immune cells and caused a 31-fold spike in antibodies compared with pre-vaccination rates with an increased response rate to Delta and other VOCs (Alpha and Beta) [127]. Contrarily, two doses of the COVID-19 vaccine candidate S-Trimer (SCB-2019) achieved a 64.2% effectiveness rate with a single dose efficacy of 49.9% [128]. Additionally, a clinical trial on NDV-HXP-S, “an inactivated egg-based Newcastle disease virus (NDV) vaccine”, against the Delta strain displayed a positive response in 84–96% of the treatment groups with an approximately four-fold increase in the neutralizing activity [129].

Real-world data collected from the United Kingdom (October 2020 to May 2021) after a mass vaccination campaign to the adult population with ChAdOx1 and BNT162b2 showed identical efficacies after a single shot, with 51.1 % and 33.5 % defense rates against the Alpha and Delta versions, respectively. Nevertheless, the prophylactic efficiency against the Delta and Alpha variants (87.9 and 93.4%, respectively), followed by double shots of BNT162b2, was pointedly higher than that of ChAdOx1 after two shots (66.1 % and 59.8 %, respectively) [130]. These findings are generally consistent with reports from Scotland. Overall, BNT162b2 outperforms ChAdOx1 in guarding against Delta and Alpha variants. Furthermore, in mainland China, during the Delta variant outburst, the defense rate of an inactivated indigenous vaccine provided 69% protection against COVID-19 contamination from close contact, 73% protection against the development of pneumonia, and more than 95 % protection against severe infection. Besides that, in phase III clinical trials, BBV152 (an India-developed inactivated vaccine) demonstrated a total prophylactic rate of 77.8% against symptomatic conditions and 65.2% against the Delta variant [61]. Hence, it should be emphasized that after two shots, the defense rates against various SARS-CoV-2 strains, in cooperation with the Delta variant, were comparatively much higher than after a single shot. Thus, raising the percentage of fully vaccinated people, in addition to increasing the completely vaccinated population, is important to successfully halt the transmission of SARS-CoV-2 globally.

### 4.2. Omicron Variants Influence Vaccine Efficacy

On November 2021, the first Omicron infection, also known as B.1.1.529, was detected in South Africa and was also reported to the WHO, which then was declared as a VOC and referred to this variant as Omicron. On comparing this variant to other known VOCs, it was found that an unusually high number of mutations (50) have been found on the spike (S) protein, the primary antigen that is targeted by antibodies produced during infection or vaccination, some of which are concerning, and preliminary evidence suggests an increased likelihood of reinfection [37,85]. Vaccines are ineffective in some high-vaccination populations due to the rapid outbreak of COVID-19 caused by this variant. Recently, a study compared vaccine effectiveness in symptomatic COVID-19 individuals infected by the Omicron and Delta variants (B.1.617.2) by administering the first two consecutive doses of the mRNA-1273 vaccine (Moderna), the BNT162b2 vaccine (Pfizer BioNTech), or the ChAdOx1 nCoV-19 vaccine (AstraZeneca), followed by a booster dose of any of the three vaccines. From this study, it was revealed that symptomatic COVID-19 caused by the Omicron variant was limited by primary vaccination with BNT162b2 or ChAdOx1nCoV-19, followed by a booster dose of mRNA-1273 or BNT162b2, which remarkably increased protection [100].

It was demonstrated that the Omicron strain may have evolved and mutated to undergo antigenic escape as a result of prolonged COVID-19 infection. According to reports, highly infectious strains such as the Omicron frequently have the N460K, S477N, and S494P mutations [93]. Despite the possibility of COVID vaccine boosters offering immunization and mRNA- or non-mRNA-based vaccinations capable of adapting to evolving variants, such as Omicron, it is concerning that a booster shot is required so soon after a full vaccination, and that additional shots may be required, given the possible health impacts. As such, there is a pressing need to develop single-dose effective vaccines that can be beneficial for people for their entire lives against Omicron and other VOIs [131]. During clinical trials and rapid deployment worldwide, SARS-CoV-2 vaccines induced strong neutralizing immunity in both humoral and cellular components and significantly reduced COVID-19 infections, hospitalizations, and deaths. Antibody epitope mutations on the spike protein can increase viral resistance and reduce vaccine effectiveness when these changes occur. As a result, monoclonal antibodies used both as a treatment and a preventive strategy against COVID-19 are strongly impaired in their activity [132,133]. 

Another group of researchers demonstrated that heterologous boosting with V-01 (recombinant SARS-CoV-2 fusion protein vaccine) consecutive to two doses of COVID-19 vaccination was 3.7 times more effective against Omicron than the homologous dose group with the same vaccine shots [134]. Additionally, a randomized clinical trial showed that a booster vaccination of CoronaVac significantly raised the geometric mean titers against Omicron and other deadly strains, with a higher level of neutralizing antibodies seen in those who received a higher dosage (6 μg) than in those who received a medium dosage (3 μg) [135]. This study was consistent with the existing literature in other populations and with different vaccines; a booster dose produced a lower titer of neutralizing antibodies against Omicron than it did against other SARS-CoV-2 strains [136]. In addition, UB-612, “a multitype subunit vaccine containing S1-RBD-sFc protein”, was shown in a phase I/II trial report to have prolonged virus-neutralizing antibodies as well as great T-cell immunity against Omicron and other COVID-causing variants, as well as a third dose, boosted immunological memory with significant antibody titers against Omicron [137]. In another study, researchers indicated that the Omicron neutralizing antibody titer was lower in convalescents and individuals who did not receive a booster dose, demonstrating that a homologous or heterologous booster could limit the ability of Omicron to evade neutralization [138].

In a recent study, the mRNA vaccines were very good at preventing COVID-19-related hospitalizations, Omicron, and other variants. However, for protection against Omicron, three doses of the vaccine were needed, while Delta and Alpha only needed two doses. Furthermore, Omicron variants were associated with less severe disease than Delta variants among adults with COVID-19, but they still resulted in significant morbidity and mortality. A marked difference in disease severity was seen between vaccinated and unvaccinated individuals among hospitalized COVID-19 patients [139]. In a most recent study, an antibody, SP1-77, was discovered to neutralize the SARS-CoV-2 Omicron variant, and this was obtained from a humanized mouse model carrying a human VH1-2 and VK1-33. This antibody has a significant complementarity-determining region 3 (CDR3) and is thus highly effective against coronavirus. SP1-77 neutralizes and binds the variants (Omicron and other VOCs) via a novel CDR3-based mode and neutralizes SARS-CoV-2 VOCs. A more direct effect of SP1-77 is that it blocks membrane fusion rather than the binding of RBD to the receptor or the endocytosis step of viral entry. Several strategies can be developed to design vaccines with robust neutralization of the current and future variants of SARS-CoV-2 based on these findings, which may lead to a more effective design of the vaccines [140,141].

## 5. Therapeutics for the Delta and Omicron Variants

COVID-19 vaccination is strongly advised as the principal option for reducing Delta variant outbreaks and the significant financial burden they impose on healthcare systems [130]. Currently, the main aim of treatment against COVID-19 is to relieve symptoms in mild to moderate cases, regardless of the type of variant. However, with the promising results of mAbs against mild to moderate COVID-19 cases, studies about the utility of the same against the Delta and Omicron variants have seen a rise among researchers [62]. A recent study suggests that treatment with mAbs can help reduce Delta COVID infection and found that three (imdevimab, etesivimab, and casirivimab) out of four (the fourth one is bamlanivimab) clinically approved monoclonal antibodies were active against the Delta variant [67]. An injection of casirivimab–imdevimab (CImAb), which is a cocktail of two monoclonal antibodies, was discovered, and this injection was intended particularly to thwart the infectivity of SARS-CoV-2 [142]. CImAb is a blend of two IgG mAbs that bind non-competitively to the RBD region of the SARS-CoV-2 spike protein and restricts the virus from interacting with the human ACE2 receptor. An in vitro study claimed that CImAb maintains its efficacy against the SARS-CoV-2 Delta variant, which led to a decrease in hospitalization rates for patients who received it [143]. Additionally, sotrovimab along with casirivimab–imdevimab is given to patients and has also proved to be effective against the Delta variant [144]. Therefore, the Delta variant and its clinical manifestations appear safe and effective when treated with mAbs [145]. According to Dr. D. Nageshwar Reddy, Chairman of AIG Hospitals in Hyderabad, monoclonal antibodies can halt the progression of the Delta variant in a patient, reducing the need for ICU hospitalizations and fatalities. A team from AIG Hospitals in Hyderabad along with researchers from “The Asian Healthcare Foundation, CCMB Hyderabad”, and the Institute of Life Sciences carried out a study where 285 patients were allocated to monoclonal antibody therapy and standard of care treatment [146]. The Delta variant was found in more than 98% of the samples analyzed. Following a week of cocktail medicine treatment, 78% of cases were symptom-free, and 100% by the end of the second week. Reddy added that 20% of patients who were treated with standard care had a more severe illness or ended up in the intensive care unit. It was found that 75% of patients who received monoclonal treatment were RT-PCR negative by the seventh day, and 78% had relief from clinical symptoms. Reddy noted that none of the research subjects became extremely ill or died, nor did they experience a rise in inflammation markers, nor did they experience post-COVID symptoms. CImAb cocktail treatment sprang to prominence after it was employed to treat then-US President Donald Trump in October of last year [147]. “The Central Drugs Standards Control Organization (CDSCO)” certified it for usage in India in May. However, it was not well-received by COVID-19 sufferers. The main cause has been the high expense. The study conducted by the team of AIG Hospitals established that when administered at the appropriate time, monoclonal treatment fully slows the course of the disease [148].

Apart from that, a research study also demonstrated the effectiveness of bamlanivimab/etesevimab—a combination of mAb for the management of COVID-19 (Delta variant) [149]. The US Centers for Disease Control and Prevention (CDC) determined that bamlanivimab and etesevimab, administered together, are expected to retain activity against the SARS-CoV-2 B.1.617.2/Delta variant. However, based on in vitro assays, bamlanivimab and etesevimab, administered together, are not expected to retain activity against the SARS-CoV-2 P.1/Gamma variant (first identified in Brazil); the B.1.351/Beta variant (first identified in South Africa), the AY.1 and AY.2 variants/Delta[+K417N] (commonly known as Delta plus, first recognized in India); and the B.1.621 variant (first identified in Colombia) [150]. Presently, three monoclonal antibody therapies—bamlanivimab and etesevimab administered together, REGEN-COV, and sotrovimab—are authorized for the treatment of mild to moderate COVID-19 in adult and pediatric patients (12 years of age and older weighing at least 40 kg) with positive results of direct SARS-CoV-2 viral testing; and who are at high risk for progression to severe COVID-19, including hospitalization or death.

The Omicron version successfully avoids the humoral immune response caused by primary immunization, despite having far more mutations than previous variations. Additional mRNA vaccine administrations, on the other hand, promote cross-neutralizing reactions toward Omicron, either through the affinity maturation of existent antibodies or through targeting novel epitopes common throughout variants [132]. Takashita et al. studied the antibodies and antiviral medicines’ effectiveness against the Omicron variant. They concluded that the susceptibility of Omicron toward molnupiravir, remdesivir, and nirmatrelvir was significantly identical to other variants of concern [151]. In 2022, Sheikh et al. in their study about the effectiveness of a booster dose against Omicron concluded that a third dose of vaccination provides substantial protection against symptomatic disease occurring due to Omicron in comparison to the second dose [152]. The development of an asymptomatic Omicron variant as an exciting live attenuated vaccine has led to the suggestion of a “virus against virus” approach as a possible solution [153]. This approach may have benefits such as exposure to structural and non-structural proteins during infection, interhost sequence diversity, and a comparatively long antigen existence in the host. However, compared with immunity induced by a naturally occurring infection, a vaccine strategy based on a single consensus version of the S protein would be more limited. The Omicron variant seems to be less infective but more contagious. Two doses of vaccination, or even a single booster shot, might not be enough to avoid infection with the Omicron variant; however, they will undoubtedly lessen the severity of the disease and the probability of death. Even so, those who are not immunized, the elderly, or those with co-existing conditions or immunosuppression are particularly vulnerable to this variant. Vaccines can nevertheless induce robust cellular and humoral immune responses, so giving people two doses and a booster shot will help protect them from an illness triggered by a coronavirus and its variants. While it has been demonstrated that with time, neutralizing antibodies diminish the maintenance of a T-cell-mediated humoral response is also a prospective protective pathway. High resistance of the Omicron variant to neutralizing antibodies evoked by existing vaccines for COVID-19, convalescent-phase sera, and therapeutic monoclonal antibodies limits the efficacy of currently available vaccines and therapies. Although booster (heterologous/homologous) vaccines are anticipated to curtail the spread of the Omicron variant, a greater insight into their durability and potency is required [138,154]. The use of Omicron-specific vaccines for boost immunization appears ineffective, whereas the incorporation of pan-sarbecovirus vaccines appears extremely promising [155,156]. The ongoing evolution of the pandemic virus with new and burgeoning variants necessitates the development of potent vaccines, which include variant-specific, mutation-resistant, and universal vaccines, to keep abreast with arising variants and to devise novel MAbs for managing COVID-19 patients and mitigating the ongoing pandemic [157].

## 6. Delta Omicron Recombinant Variant

SARS-CoV-2 is susceptible to genetic changes over time, much like other viruses; it can exert a slight or major influence on its infectiousness, characteristics, and pathogenicity. The genetic alterations might result in the aggregation of mutations, greater transmissibility, and a shift in therapeutic vaccination efficacy. Mutations, insertions, and recombination are all examples of genetic alterations. Moreover, the tracking of the recombinant viruses may thus aid in the preparation for these occurrences, as well as the optimization of treatment and preventative techniques [158]. Recombination is a technique for a virus to develop a different mutation combination. In humans, the first 87,695 genomes of SARS-CoV-2 posted on 2021′s GISAID revealed 225 sequences with plausible recombinant provenance [159]. Despite the publication of various data on the recombination of the Alpha and Delta variants of SARS-CoV-2, the data available for the recombination of Delta and Omicron variants are very small. In 2022, Lacek et al. claimed to have discovered the first SARS-CoV-2 recombinant genome consisting of a hybridized spike protein obtained from an Omicron (BA.1.1)–Delta (AY.119.2) recombination incident [160]. According to the researchers, the recombinant resembles the Delta variant (AY.4) except for the region encoding the spike gene, which resembles BA.1. 27 amino acid mutations were found in BA.1, five in AY.4, and four in both [161]. In another study conducted by Bolze et al. in 2022, the team sequenced 29,719 positive samples acquired between November 2021 and February 2022, when both Omicron and Delta remained co-circulated in the US. They observed 20 co-infections and 2 separate incidences of infection by the recombinant Delta-Omicron virus. They stated that such recombinants appeared uncommon and that there was no indication that the ones found in this investigation constituted more transmissibility than the Omicron lineages that were already circulating (BA.1, BA.2) [162].

## 7. Concluding Remarks and Future Prospects

Before the discovery of the Omicron variant, the Delta variant of SARS-CoV-2 was considered the most infectious form of the virus, being 40–60% more infectious than the Alpha variant. Moreover, the Delta variant is nearly two-fold more infectious than the initial Wuhan strain and contains considerably more viral particles in the airways of patients. A Chinese study revealed the virus concentrations in Delta infections were 1000-fold greater in comparison with other variants [163]. Following this report, the WHO declared the Delta variant to be the “fastest and fittest” version of SARS-CoV-2 [164]. However, recently, Omicron has been identified as a more transmissible strain, along with a high infectivity rate and decreased vaccine effectiveness, than Delta ever was due to multiple unique mutations on its S protein and RBD [165]. Two doses of vaccine are beneficial in reducing hospitalization and mortality, though the neutralization titer of vaccinated serum is lower against the Delta mutant than the original strain [164]. When developing booster immunization guidelines, the cumulative impacts of limited neutralizing antibody levels due to both age and the VOC must be recognized [166]. The original study constraints included a small number of subjects and the likelihood of unrecognized infectious disease prior to immunization. Collectively, various types of research highlight the necessity of complete SARS-CoV-2 vaccination, although reports of decreased vaccine efficacy against Delta necessitate more research into breakthrough infections, along with the prospect of booster vaccine doses [167,168]. Already, to improve efficacy against circulating variants, booster dosages are being developed by some companies. Pfizer aims to obtain FDA clearance for their booster dose, which is anticipated to neutralize the Delta variant more effectively [169]. COVID-19 prevalence surged worldwide, with an average of almost four lakh cases reported every day, up from 370,000 per day the week before. The overall number of documented cases has already surpassed 186 million, with nearly 4 million fatalities. Furthermore, there are still deficiencies in epidemiological monitoring, testing, and genomic sequencing in many parts of the globe, limiting our potential to track and analyze the effects of existing and future mutations promptly. As most of the newly surfaced VOCs have higher transmissibility and evasiveness, nasal vaccination is also the better option on which many firms are working, as it provides localized IgA-mediated immunity to prevent infection [12].

The importance of immunizations in saving lives (preventing serious illness) was not instilled in people. The lack of information on major adverse outcomes following immunization exacerbated people’s concerns. Furthermore, many vaccine-related deaths have been reported in the media without a justification for the trigger, leading to widespread skepticism. As a consequence of the lack of trustworthiness, misconceptions have been compounded in scientific research, as the evidence was not obvious. The most significant method for preventing and controlling the spread of the Delta and Omicron strains is to increase vaccination coverage. The pervasive nature of the Delta variant necessitates the worldwide augmentation of the vaccination process. Accelerating research and development in the field of vaccines is of utmost importance. The FDA, WHO, EMA, and the UK have all issued specific recommendation guidelines for vaccination research and development focusing on variants. However, more research is required to understand the strain’s distinct symptoms along with invasion patterns. Presumably, this newfound understanding will aid in the development of therapies and various prophylactic methods, such as vaccination campaigns and other public initiatives.

## Figures and Tables

**Table 2:** Characteristic mutations of Omicron variants [90].

Sub Variants	Gene	Amino Acid Mutation	Mutation Impact	Omicron Variant Lineages
21K (Omicron)	S	E484A	In other variants, mutations to this amino acid have been linked with antigenic escape, as well as mutations to other amino acids at this position [91].	BA.1
		H655Y, N679K, P681H	May increase transmissibility [92].	
		Q498R, N501Y	The binding to the ACE2 is increased by a significant amount [93].	
		A67V, T95I, Y145D, H69, L212I, G339D, S371L, S373P, V70, S375F, K417N, N440K, G446S, G142, S477N, T478K, Q493R, G496S, V143, N501Y, Y505H, T547K, D614G, Y144, N764K, D796Y, N856K, Q954H, N211, N969K, L981F		
	N	P13L		
		R203K, G204R	Viral loads and subgenomic RNA expression are increased [94,95].	
		E31-, R32-, S33-		
	ORF1a	K856R, S2083-, L2084I, I3758V, A2710T, T3255I, P3395H		
		G3676, L3674, S3675	These mutations have been hypothesized to promote the evasion of innate immunity by impairing cells’ capacity to break down components of the virus [96].	
	ORF1B	P314L, I1566V		
	ORF9b	P10S, E27-, N28-, A29-	By interacting with TOM70 and NEMO, ORF9b suppresses the innate immune response to infection, resulting in the generation of IFN [97,98].	
	E	T91I		
	M	D3, Q19, A63T		
21L (Omicron)	S	T19I, V213G, T376A, D405N, S371F, R408S: (6 additional spike mutations) A27S, G142D, G339D, S373P, L24, S375F, K417N, N440K, P25, S477N, T478K, E484A, P26, Y505H, Q493R, Q498R, N501Y, N969K, D614G, H655Y, N679K, Q954H, P681H, N764K, D796Y		BA.2
	N	P13L, E31, S413R, R32, S33, R203K, G204R		
	ORF1a	L3201F	May have originated in South Africa but it is more common in other countries than in the country of origin.	
		S135R, T842I, G1307S, F3677, L3027F, T3090I, T3255I, G3676, P3395H, S3675		
	ORF1b	P314L, T2163I, R1315C, I1566V		
	ORF3a	T223I		
	ORF6	D61L		
	ORF9b	P10S, A29, E27, N28		
	E	T9I		
	M	Q19E, A63T		
22A (Omicron)	S	F486V, R493Q	Due to the mutation and reversion, binding affinity to ACE2 is reduced [89].	BA.4
		T19I, L24, N969K, P25, P26, Q954H, A27S, H69, D796Y, V70, N764K, G339D, S371F, P681H, S373P, S375F, N679K, T376A, D405N, H655Y, R408S, K417N, D614G, N440K, L452R, S477N, Y505H, T478K, E484A, F486V, Q498R, N501Y,		
	N	S413R, P13L, E31, G204R, R32, S33, P151S, R203K		
	ORF1a	F3677, S135R, K141, G3676, S142, F143, S3675, T842I, G1307S, P3395H, L3027F, T3090I, T3255I		
	ORF1b	P314L, T2163I, R1315C, I1566V		
	ORF3a	T223I		
	ORF6	D61L		
	ORF7b	L11F		
	ORF9b	P10S, E27-, N28-, A29-		
	E	T9I		
	M	Q19E, A63T		
22B (Omicron)	S	T19I, L24-, N969K, P25-, P26-, Q954H, A27S, H69-, D796Y, V70-, G142D, V213G, N764K, G339D, S371F, P681H, S373P, S375F, T376A, N679K, D405N, R408S, H655Y, D614G, K417N, N440K, L452R, S477N, Y505H, T478K, E484A, F486V, N501Y, Q498R	A reversion at R493Q and the F486V mutation may have caused the marked reduction in ACE2 binding affinity in 22A (Omicron) and 22B (Omicron) compared to 21K (Omicron) [89].	BA.5
	N	P13L, E31, S413R, R32-, S33-, G204R, R203K		
	ORF1a	S135R, T842I, F3677, G1307S, L3027F, G3676, T3090I, T3255I, S3675, P3395H		
	ORF1b	P314L, T2163I, R1315C, I1566V		
	ORF3a	T223I		
	ORF9b	P10S, A29, E27, N28		
	E	T9I		
	M	A63T, D3N, Q19E		
22C (Omicron)	S	N969K, T19I, L24, Q954H, P25, P26, A27S, D796Y, G142D, V213G, N764K, G339D, S704L, S371F, S373P, P681H, S375F, T376A, N679K, D405N, R408S, K417N, H655Y, N440K, L452Q, D614G, S477N, T478K, Y505H, E484A, Q493R, Q498R, N501Y		BA.2.12.1
	N	P13L, E31, G204R, S413R, R32, R203K, S33		
	ORF1a	S135R, T842I, F3677, G1307S, L3027F, G3676, T3090I, L3201F, S3675, T3255I, P3395H	L3201 originated in South Africa as a wild type but it is common in other countries.	
	ORF1b	P314L, R1315C, T2163I, I1566V		
	ORF3a	T223I		
	ORF6	D61L		
	ORF9b	P10S, A29, E27, N28		
	E	T9I		
	M	Q19E, A63T		
22D (Omicron)	S	T19I, L24, N969K, P25, P26, Q954H, A27S, D796Y, G142D, N764K, K147E, D796K, P681H, W152R, N679K, F157L, I210V, H655Y, I210V, V213G, D614G, G257S, G339H, Y505H, SS373P, S375F, N501Y, T376A, D405N, Q498R, R408S, K417N, R493Q, N440K, G446S, E484A, N460K, S477N, T478K	Mutations in N460K, G446S, G339H, and R493Q may cause 21L(Omicron)-induced immunity to be overcome. May also have higher ACE-2 binding affinity than 22A (Omicron)/22B (Omicron) [89].	BA.2.75
	N	P13L, E31, S413R, R32, S33, R203K, G204R		
	ORF1a	S135R, T842I, N4060S, S1221L, G1307S, F3677, P1640S, L3027F, G3676, T3090I, L3201F, S3675, T3255I, P3395H		
	ORF1b	P314L, G662S, T2163I, R1315C, I1566V		
	ORF3a	T223I		
	ORF6	D61L		
	ORF9b	P10S, A29, E27, N28		
	E	T9I, T11A		
	M	Q19E, A63T		

S = spike protein, ORF = open reading frames, N = nucleocapsid, M = membrane protein, E = envelope, amino acid- = deletion of the mentioned amino acid, e.g., H69-.

## Data Availability

Not applicable.
